# Palliative Care in Severe Neurotrauma Patients in the Intensive Care Unit

**DOI:** 10.1007/s12028-023-01717-1

**Published:** 2023-05-12

**Authors:** Rianne G. F. Dolmans, Faith C. Robertson, Marleen Eijkholt, Peter van Vliet, Marike L. D. Broekman

**Affiliations:** 1 Department of Neurosurgery, Leiden University Medical Center, Albinusdreef 2, 2333 ZA Leiden, The Netherlands; 2 Department of Neurosurgery, Massachusetts General Hospital, Boston, MA USA; 3 Department of Ethics and Law in Healthcare, Leiden University Medical Center, Leiden, The Netherlands; 4 Department of Intensive Care Medicine, Haaglanden Medical Centre, The Hague, The Netherlands; 5 Department of Neurosurgery, Haaglanden Medical Centre, The Hague, The Netherlands

**Keywords:** Traumatic brain injury, Neurotrauma, Palliative care, Neurocritical care

## Abstract

Traumatic brain injury (TBI) is a significant cause of mortality and morbidity worldwide and many patients with TBI require intensive care unit (ICU) management. When facing a life-threatening illness, such as TBI, a palliative care approach that focuses on noncurative aspects of care should always be considered in the ICU. Research shows that neurosurgical patients in the ICU receive palliative care less frequently than the medical patients in the ICU, which is a missed opportunity for these patients. However, providing appropriate palliative care to neurotrauma patients in an ICU can be difficult, particularly for young adult patients. The patients’ prognoses are often unclear, the likelihood of advance directives is small, and the bereaved families must act as decision-makers. This article highlights the different aspects of the palliative care approach as well as barriers and challenges that accompany the TBI patient population, with a particular focus on young adult patients with TBI and the role of their family members. The article concludes with recommendations for physicians for effective and adequate communication to successfully implement the palliative care approach into standard ICU care and to improve quality of care for patients with TBI and their families.

## Introduction

Traumatic brain injury (TBI) remains a significant cause of mortality and morbidity worldwide [1]. In the United States alone, there are approximately 2.8 million TBI-related emergency department visits per year, with about 56,000 of those resulting in death [2]. Roughly 10–15% of patients with TBI have serious injuries that require intensive care unit (ICU) management [3]. Peak incidences of TBI occur in children during early childhood, in young adults, and in the elderly [1, 4], and a significant number of these extremely vulnerable groups requires ICU management.

Intensive care management of patients with TBI has unique challenges, particularly in the setting of young adult patients. Uncertain prognosis, no advance directives, and bereaved families that must act as decision-makers can make ICU care difficult. Symptom management, treatment aimed at recovery, and goals of care discussions in relation to the patients’ wishes are foundational to ICU care [5]. When facing a life-threatening illness such as TBI, palliative care is equally important as standard ICU care and should always be considered in patients with TBI in the ICU. However, research shows that palliative care is used less frequently in neurology and neurosurgery patients in the ICU [6–8]. This finding is pivotal because the status quo involves missed opportunities for these patients.

Palliative care is both a philosophy and an organized, highly structured approach for providing care to patients and their families facing a life-threatening illness, beyond imminent death [9–11]. It focuses on and dedicates time to non-curative-focused aspects of care, including physical, psychological, cultural, social, spiritual, religious, ethical, and legal aspects of care [12, 13], which we will explain in detail later on in this article. The palliative care approach can be provided together with life-prolonging care or as the main focus of care [11]. Hereafter, we use the terms “palliative care” and “palliative care approach” interchangeably. The goal of palliative care is to ensure the best possible quality of life for patients and their families, regardless of the stage of the disease or the need for concurrent therapies [10, 11]. The palliative care approach requires a specific attitude and some investment of time. The attitude and way of care can be adopted by a specialized palliative care team and by the general ICU team [10, 12].

This article will highlight the different aspects of a palliative care approach as well as the barriers and opportunities in patients with TBI in the ICU, with a particular focus on young adult patients with TBI and the role of their family members. This article also outlines skills that may aid critical care teams to implement a palliative care approach and thus improve quality of care for patients and their families.

## Palliative Care as an Approach That Should Always be Considered for Patients with TBI in the ICU

Health care providers are often hesitant to incorporate a palliative care approach because of misconceptions around palliative care [14]. Historically, the delay in a palliative care approach has resulted from assumptions that palliative care is associated with oncology, hospice care, or end-of-life situations.

The biased perception of palliative care as an end-of life service leads providers to categorize it as a separate pathway rather than a complementary option. Consequently, palliative care is not integrated simultaneously with curative treatment as often as it could be [13, 15–17]. This is one of the reasons why there is limited use of palliative care in (neuro)trauma patients [16, 18].

Palliative care is often not initiated until after the decision to withdraw lifesaving measures, as seen in the Collaborative European NeuroTrauma Effectiveness Research in Traumatic Brain Injury (CENTER-TBI) study, in which 79% of patients with TBI had palliative care initiated after that point [19]. There is variability by location and culture regarding initiation of palliative care. In northern Europe, southern Europe, and western Europe, palliative care was initiated in 78%, 92%, and 96% of centers, respectively, which is in contrast to the Baltic States, where palliative care was not initiated in 60% of the centers [19]. Not only in Europe but also in the United States, it is found that palliative care is often not integrated in the care of patients with TBI. A study by Kross et al. examining whether ICU attending specialty was associated with quality of end-of-life care found that neurosurgical patients and neurology patients in the ICU received palliative care less frequently than medical patients in the ICU [6]. Although the use of palliative care has increased since the study conducted by Kross et al. (2003–2008), more recent studies still show that palliative care is used less frequently in neurology and neurosurgery patients. For example, Hwang et al. found that, in 5,733 older (> 55 years) patients with TBI, only 35% of patients received a palliative care approach during hospitalization [8]. However, it must be noted that this article used the *International Classification of Diseases, Ninth Revision* code V66.7, which stands for “palliative care encounter,” which encompasses not only end-of-life care and terminal care aimed at relieving pain and discomfort but also hospice care. Thus, the number of patients who received a palliative care approach as discussed in the current article might be even lower given the limitation of the methods used to identify the palliative care approach. This is also evident from a survey study from Neurocritical Care Society members that found that a palliative care approach was only used in < 11% of neuro-ICU patients [7].

Integrating a palliative care approach can provide structure and helpful insights within high-intensity care plans. Such approach can facilitate earlier consensus around non-curative-focused aspects of care and proxy-decision-makers and may also include end-of-life decisions. A palliative care approach would improve quality of care, decrease preterminal ICU days for the dying patient, and decrease total hospital costs [8, 20–25]. We submit that for patients with TBI in the critical care setting, a palliative care approach should always be considered. Given high mortality rates in patients with TBI, a palliative care approach for this group would seem particularly relevant. This ambition aligns with the expanding field of palliative care; the palliative care field has increased application for additional patient populations, such as patients with lung diseases and neurologic diseases as well as trauma patients [10, 12, 26, 27].

## Different Aspects of the Palliative Care Approach in Neurotrauma Patients

As stated, the palliative care approach encompasses several areas of care and can be provided by a specialized palliative care team and by the general ICU team, depending on the hospital. Below we describe some of the different aspects of the palliative care approach specific for this patient group and their context.

### Physical Aspects of Care

The physical aspects of care are mostly focused on symptom management using best practices to reduce symptom burden with treatment to a level acceptable to the patient and to provide optimal patient comfort [28]. Pain is the most prevalent and distressing physical symptom in critically ill patients [28, 29]., However, uncertainty exists if patients with severe brain injury have incomplete pain perception [30]. Because patients with severe TBI are often sedated and intubated in the ICU, there is minimal ability to communicate their symptoms. Thus, we rely on validated tools, such as the Behavioral Pain Scale, the Brain Injury Nociception Assessment Measure, and the Critical Care Pain Observation Tools, that incorporate facial expressions, body movement, and compliance with mechanical ventilation [30–32]. In addition, signs of sympathetic activation can be noted when a patient is in discomfort, such as hypertension, tachycardia, tachypnea, diaphoresis, and piloerection [31]. Based on these findings, sedation and/or analgesia can be adjusted as necessary by the critical care team. For family members, reducing symptom burden and seeing that the patient is comfortable is important for their coping strategy and to have confidence in the way the patient is treated.

### Psychological Aspects of Care

Psychological symptom management is a key aspect to palliative care in critically ill patients because they frequently experience psychological symptoms, such as confusion, anxiety, and depression [33]. However, severely injured neurotrauma patients are often deeply sedated, which leads to the misperception that palliative care approaches are not an immediate concern. In neurotrauma patients, this psychological aspect of care should therefore also, at least initially, focus on family members. The well-being of family members is often overlooked by health care workers, and family members are often referred to as “hidden patients” [34]. A study by Kristjanson et al. found that family members’ health deteriorates during the palliative care phase as well as during the bereavement period [34]. In addition, family members have different coping mechanisms, varying from seeking support from one another to expressing distressing emotions and adjusting expectations to denial and self-blame [35]. It is important to keep this in mind and give family members room to express their feelings. This aspect of care can be provided by the general ICU team and by a specialized palliative care team. A social worker can be assigned to the family if necessary. After all, memories about the care experience play an important role in the process of grief in the bereavement period [35, 36].

### Social Aspects of Care

Social aspects of palliative care include attention to the possible changing roles and responsibilities of family members. This includes possible financial and/or insurance problems as well as the coping process of the patients’ relatives [35, 37]. With uncertain prognoses, family members might have worries related to these aspects. The care providers can support and discuss the findings with the patients’ relatives and can take appropriate and preventive measures. If necessary, the care providers can refer the family members to the appropriate services.

### Cultural, Spiritual, and Religious Aspects of Care

In the care of neurotrauma patients, patients and their families are confronted with the finiteness of existence. This realization can significantly impact one’s state of mind and is especially deserving of health care providers’ attention because this impacts decisions about the medical care [34, 35]. It is important to pay attention to spirituality with every patient and to show interest with an open listening attitude and acknowledge and tailor the attention to the need of the family members because such attention is imperative in all parts of a life-limiting disease.

Cultural differences can be particularly pressing in a life-is-finite context, including in end-of-life phases. This equally merits attention under the palliative care approach. Different cultures can have different ways of looking at life, hope, and death, including the rituals around these phenomena [38]. The palliative care approach can be an important tool to acknowledge these differences and give family members room for their cultural habits and rituals. A spiritual care team or chaplaincy can be offered to make it easier for family members to discuss and cope with the situation in their own language and culture.

### Ethical and Legal Aspects of Care

Implementing a palliative care approach may stumble on dilemmas around proxy-decision-makers and/or pain control. It is important that caregivers recognize these moral dilemmas and support patients or family members to explore these issues together or independent of the health care providers. A palliative care approach is often offered in areas where moral dilemmas exist regarding resuscitation, mechanical ventilation, and withholding and withdrawing treatments [39]. Such issues might be exacerbated in patients with TBI, in whom the prognosis can be uncertain. Caregivers ideally have awareness of the issues but also of the structures to address these issues. When the providers are part of these issues, it seems imperative to create opportunities to conduct in-depth moral deliberations with colleagues, for example, or with ethics services. In decisions about withdrawal of life-supporting care, multidisciplinary discussions would ideally be achieved, and families should be included in discussions about which approach is considered best [40].

In summary, the different aspects of a palliative care approach in patients with severe TBI in the ICU should focus not only on the patient but also on the family. It is critical to involve them in all aspects of the palliative care approach, and depending on institutional resources, this may be steered by a specialized palliative care team and by the general ICU team.

## Barriers and Challenges in Palliative Care in Neurotrauma Patients

**Fig. 1:**
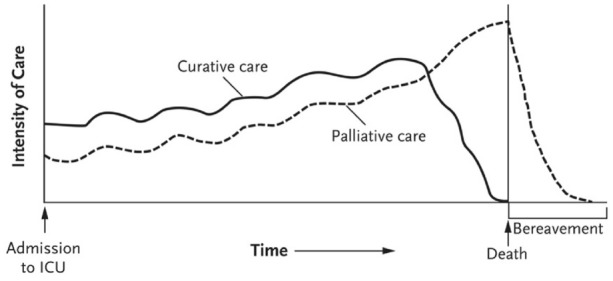
The intensity of curative and palliative care in the intensive care unit. ICU intensive care unit. From: Cook et al. [15]

One of the barriers in integrating a palliative care approach is the misconception about the nature of palliative care, as stated previously. However, there are several other barriers for integrating a palliative care approach specifically in neurotrauma patients. Uncertainty regarding prognosis for patients with severe TBI is a leading barrier to palliative care, particularly immediately after the injury, when neurologic exams are clouded by the need for sedation [25, 41]. With uncertain prognosis, most critical care teams tend to postpone implementing a palliative care approach because of the common misconception that starting with palliative care means that the patient is going to die soon [42]. However, the palliative care approach is an important part of critical care management, regardless of whether the medical condition is in an early or late stage [15] (Fig. 1).

In addition to an uncertain prognosis, another barrier in implementing a palliative care approach is that health care workers assume that people talk to their loved ones about what they do and do not want in medical procedures or about the way they would want to live their lives. However, research has shown that not many patients have discussed their wishes with their loved ones [18, 43]. Especially because TBI may happen at any moment in a previously healthy individual, advance directives or other forms of legal directives are often not present [44]. A study by Rao et al. investigated the presence of advance directives in the adult population and found that only 26.3% of US adults had advance directives [43]. Moreover, most advance directives are not readily available when needed, and instructional advance directives are often not sufficiently clear and detailed to apply to the patient’s current situation [40, 44]. Consequently, it is often unclear what the patient would have wanted, resulting in the continuation of aggressive care measures that are not primarily focused on quality of life. This can result in delays around quality-of-life measures and noncurative aspects of care or in delays and disagreement about what the trajectory toward death should look like. Implementing a palliative care approach early on during admission to focus on life-is-finite components might be helpful to avoid such delays in appropriate care.

Aside from the aforementioned obstacles that might exist in integrating a palliative care approach, there are also several barriers in offering palliative care.

One of these barriers is that the primary communication in patients with severe TBI will be with the patients’ family members and/or loved ones instead of with the patients themselves. Conflict between family members or between families and medical staff occurs often in medical care, and a palliative care setting may be particularly vulnerable [45]. In a study at six intensive care units at a medical center in the United States, 406 physicians and nurses were interviewed about conflict in an ICU in patients in whom withdrawal or withholding of treatment was considered. They found that conflict within the family occurred in 24% of cases [46]. A conflict within families can have a direct impact on patient care when clinicians rely on the patient’s family members in decision-making. Familial conflict about decision-making can lead to prolongation of dying or it can result in care that the patient did not want [47]. In addition, family conflict can also influence the grieving process in the bereavement period [48–50]. It is important to differentiate health care–related family conflicts from broader family dynamics and to discuss previous medical decision-making experiences with family members [51].

Further complicating the decision-making procedures is that the surrogates, often family members, are also in a phase of grief: grief about the uncertain prognosis of the patient and about whether the patient will ever be able to function in the same role as before as well as about the possible death of the patient. Grief and its associated emotions can complicate and often interfere with effective communication and optimized care strategies [52, 53].

Lastly, in our multicultural world, there are diverse cultural and religious traditions within society that are challenging in the medical world [54], which might be particularly relevant in the palliative care setting. One example of this, specifically in patients with severe TBI, is that brain death can occur. The state of brain death is widely, but not universally, accepted [55]. Religion plays an important role in the acceptance of brain death [56, 57]. When the family and the critical care team do not agree on this, it can be difficult to discuss implementing a palliative care approach. As discussed before, clear communication and mutual understanding are of paramount importance in providing the optimal care routes.

Besides these barriers that are specifically relevant for neurotrauma patients, there are also general barriers for integrating a palliative care approach, such as lack of care coordination, limited time, excessive paperwork, and a lack of knowledge and training about palliative care [14, 58, 59].

## Implementing a Palliative Care Approach

Despite these challenges, there are many opportunities to improve the palliative care approach in this patient group. A prospective observational pre-post study by Mosenthal et al. [23] found that integration of a structured palliative care program into standard critical care practice led to enhancement of communication between physicians, nurses, and family, with more discussions about goals of care, earlier consensus around life-supporting treatments, and end-of-life decisions. It was associated with improved outcome and reduced hospital length of stay (7.6 vs. 6.1 days) in trauma patients, including neurotrauma patients at a level I trauma center ICU [23]. The structured palliative care program consisted of two parts. Part I included early (at admission) family bereavement support, assessment of prognosis, and patient preferences. Part II included an interdisciplinary family meeting within 72 h. Another study conducted by O’Mahony et al. found similar results in which integration of a specialized palliative care team consisting of an advance practice nurse, a palliative medicine physician, and a social worker into standard ICU care was associated with improved quality of care and higher rates of formalization of advance directives, such as do-not-resuscitate orders (33.0% vs. 83.4%), as well as a decrease in the use of certain nonbeneficial life-prolonging treatments [20]. A study by Norton et al. in medical ICUs also found that integrating a palliative care approach in the ICU was associated with shorter ICU length of stay, with no significant changes in mortality rates (8.96 vs. 16.28 days) [21]. These studies emphasize the benefits of a systematic approach to palliative care in critically ill patients, including patients with TBI, in the ICU.

## Communication Skills for the Physician

Communication skills is a major part of all aspects of palliative care [18, 60, 61]. If the critical care team needs to provide palliative care, it is important that ICU staff are trained in effective and adequate communication that can cover all the different aspects of palliative care. Several studies have shown the benefits of such targeted training methods, including improved communication skills, improved methods of delivering bad news, improved recognition and management of emotional reactions, and improved techniques to convey compassion [51, 62–64].

Family members of ICU patients are important stakeholders in the palliative care approach, and they also rate communication as one of the most important needs [65–67]. The suggestion is that once the decision has been made to transition into end-of-life care and comfort measurements, the focus shifts to communication with the family members and their needs. However, a study by Curtis et al. showed that ICU physicians missed opportunities to give information or provide support in 29% of family meetings [61]. From these and other studies, we offer some suggestions on communication in the context of ICU-admitted patients with TBI that could assist in palliative care.

Communicating clearly to the family about what happened to the patient, what treatments are given, and what the outcome will be is of paramount importance for family members to understand what is happening [41]. Seaman et al. have proposed five major goals of clinician–family communication in the ICU setting based on extensive review of the literature [68]. The five goals are (1) establishing a trusting relationship between the family and physician; (2) providing emotional support to families; (3) helping families to understand diagnosis, prognosis, and treatment options; (4) allowing clinicians to understand the patients as a person; and (5) creating conditions for careful deliberation about difficult decisions.

**Fig. 2:**
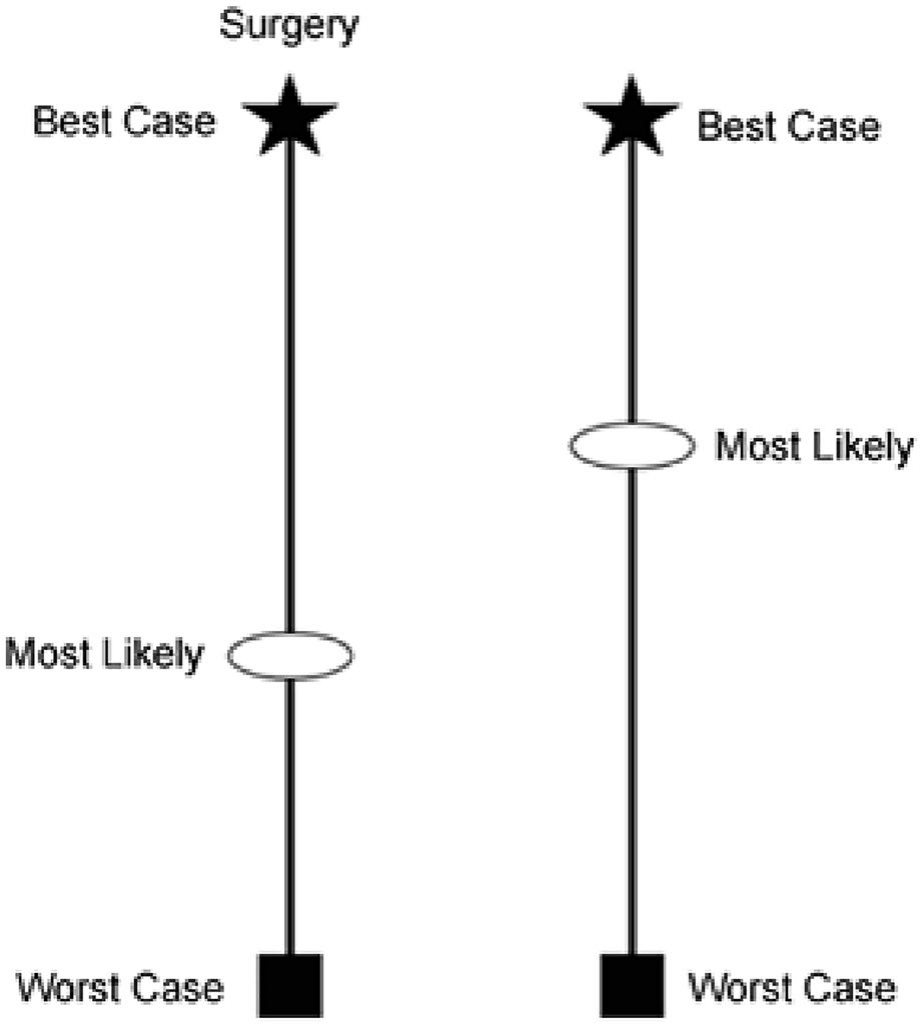
Best case/worst case tool. The star represents the best-case outcome, and the square represents the worst-case outcome. The circle represents the physician’s best judgement about the patient’s outcome. From: Taylor et al. [70]

Several communication frameworks have been developed for a systematic approach to patient and family communication and to achieve the beforementioned goals. One of these frameworks is the “best case/worst case” [69, 70]. This framework can be used to describe the best and worst possible outcomes of each treatment option, starting from where the patient is now, and to discuss what is possible [69] (Fig. 2).

In this communication trajectory, there should be sufficient attention for family members’ grief. Grief is a natural process to the loss of a loved one. The grieving process varies per individual, and bereavement support varies according to the needs of the individual [71]. The loss of a loved one can be very overwhelming, and questions about the course of the ICU stay often arise later, when the loss has been processed. Therefore, a follow-up conversation after a few weeks can be very valuable for family members and would ideally be part and parcel of a palliative care trajectory.

## Conclusions

Providing appropriate palliative care to patients with TBI in an intensive care setting can be difficult, particularly for young adult patients. Uncertainty regarding prognosis, the absence of advance directives, and the inability to talk to the patient directly can be barriers to adopting a palliative care approach. However, integration of the palliative care approach into usual critical care can lead to earlier consensus around life-supporting treatments and end-of-life decisions, improve quality of care, and decrease preterminal ICU days for the dying patient. Important factors in adequate palliative care are communication and leveraging multidisciplinary teams. We recommend that institutions review their internal policies and processes for palliative care in the ICU setting and work toward earlier integration of palliative care practices in neurotrauma patients.
